# Hydrologic Remote Sensing and Land Surface Data Assimilation

**DOI:** 10.3390/s8052986

**Published:** 2008-05-06

**Authors:** Hamid Moradkhani

**Affiliations:** Portland State University, Department of Civil and Environmental Engineering, 1930 SW 4^th^ Ave. suite 200, Portland, Oregon 97201, USA; Phone +1-503-725-2436, Fax +1-503-725-5950; Email: hamidm@cecs.pdx.edu

**Keywords:** Remote sensing, Soil Moisture, Snow, Data Assimilation

## Abstract

Accurate, reliable and skillful forecasting of key environmental variables such as soil moisture and snow are of paramount importance due to their strong influence on many water resources applications including flood control, agricultural production and effective water resources management which collectively control the behavior of the climate system. Soil moisture is a key state variable in land surface–atmosphere interactions affecting surface energy fluxes, runoff and the radiation balance. Snow processes also have a large influence on land-atmosphere energy exchanges due to snow high albedo, low thermal conductivity and considerable spatial and temporal variability resulting in the dramatic change on surface and ground temperature. Measurement of these two variables is possible through variety of methods using ground-based and remote sensing procedures. Remote sensing, however, holds great promise for soil moisture and snow measurements which have considerable spatial and temporal variability. Merging these measurements with hydrologic model outputs in a systematic and effective way results in an improvement of land surface model prediction. Data Assimilation provides a mechanism to combine these two sources of estimation. Much success has been attained in recent years in using data from passive microwave sensors and assimilating them into the models. This paper provides an overview of the remote sensing measurement techniques for soil moisture and snow data and describes the advances in data assimilation techniques through the ensemble filtering, mainly Ensemble Kalman filter (EnKF) and Particle filter (PF), for improving the model prediction and reducing the uncertainties involved in prediction process. It is believed that PF provides a complete representation of the probability distribution of state variables of interests (according to sequential Bayes law) and could be a strong alternative to EnKF which is subject to some limitations including the linear updating rule and assumption of jointly normal distribution of errors in state variables and observation.

## 1. Introduction

Hydrologic modeling has greatly benefited from observation of land surface water, energy, and carbon conditions which are of critical importance owing to their profound impacts on real world water resources applications such as flood control, weather and climate prediction, agricultural production and water resources management which collectively control the behavior of the climate system.

Many studies have demonstrated that initial and boundary conditions of state variables such as soil moisture, soil temperature or vegetation water content at different temporal and spatial scales exercise strong controls on climate, weather and hydrologic processes. [9, 11, 21, 59]. Observing these state variables and assimilating them into hydrologic models to improve the model prediction are crucial for natural resources management, flood forecasting, and crop management. Depending on the spatial scale of interest, there are different ways to measure these state variables. At the local scale, in-situ techniques provide fairly accurate measurements of the state variables at the time scale of interest. If in-situ observations are directly incorporated and used in large scale models they pose limitations due to their very small spatial support. An alternative would be the incorporation of satellite remotely-sensed measurements which provide spatially integrated measurement of state variables with a specific temporal sampling depending upon the orbital placement of the satellites.

Remote sensing has shown great promise for providing an abundance of data and information that were lacking with the in-situ observations. It has also been a valuable tool in many hydrologic modeling applications due to its capability of providing unrestricted collection of information with wide spatial coverage and temporal repeat [31].

Soil moisture plays a key role in the terrestrial water cycle and is responsible for the partitioning of precipitation between surface water (runoff) and storage through infiltration. Surface and root zone soil moisture control the redistribution of incoming radiation (available energy) on the land surface into sensible and latent heat (evaporative) fluxes. Understanding soil moisture is pivotal in various fields such as agriculture, ecology, hydrology and even geotechnical engineering. Furthermore, root zone soil moisture carries memory from weekly to monthly timescales; therefore its accurate initialization may contribute to enhanced prediction of summer precipitation [14, 18, 36]. Soil moisture regulates the availability of water and nutrients to plants and it has a significant impact on global water cycles. The change in the meteorological fluxes that drive soil moisture is subject to large-scale variations in soil moisture creating a feedback mechanism that can have considerable influence on climate and land use change [3, 20, 23].

Snowpack is a major component of seasonal water supply in many middle to high latitude alpine catchments and it contributes a considerable percentage (for example, 70-80 percent in the northwestern US) to the total annual runoff in these regions. Snow has large influence on land-atmosphere energy exchanges due to its high albedo, low thermal conductivity and considerable spatial and temporal variability resulting in the dramatic change of surface and ground temperature. Accurate estimation of the amount and timing of snowmelt coupled with proper monitoring of snow properties including snow extent and snow water equivalent (SWE) are vital to estimating a more accurate water supply forecast as required for water resources management. Analyses of snowpack observations collected over the past decade indicate that packs are melting earlier in the year and SWE data collected from snow course sites display negative trends over the period of record from 1950-2000. Therefore, understanding the evolution of snow energy and mass balance processes is imperative for a complete description of the hydrological cycle at basin and regional scales to accurately characterize and estimate snow properties in the snow dominated regions for flood and avalanche warnings, environmental compliance, and water geochemistry for use by water resources managers and planers.

## 2. Soil Moisture Observation

Soil moisture information may be obtained in two ways: 1) it may be derived by running a land surface model through which the meteorological forcing observation is propagated; 2) it may be retrieved from in-situ measurement or from low-frequency passive and active microwave data. It has long been recognized that reliable, robust and automated methods for the measurement of soil moisture content could be extremely useful, if not essential, in hydrologic, environmental and agricultural applications. Despite the availability of various methods in retrieving soil moisture at a single location there are currently no networks of in-situ sensors that provide regional or global data sets. Considering that such networks are expensive and impracticable, attention has gone to remote sensing data, which are able to provide large-scale information suitable for regional and global applications. Platforms for supporting remote sensing instruments have varied from ground-based supports to aircraft and satellites. Ground-based systems can be mounted on trucks or on special structures such as rails to allow for movement of the sensor. The advantage of these ground-based systems is the relatively small footprint of the sensor providing easy control during the measurement period. The main disadvantage is the small coverage of large areas. The aircraft mounted systems can overcome some of these limitations while mapping the larger area and can serve as prototypes for future satellite sensors. However, satellite remote sensing offers the optimal solution owing to their capability of monitoring large areas with long term repetitive coverage.

Satellite observations alone are not sufficient because of the temporal and spatial gaps in their coverage. Also the deeper soil moistures cannot be observed directly from space. Therefore, the best possible system would integrate the benefits of land surface models, in-situ and satellite observations to assess global soil moisture conditions. This can be done through Data Assimilation (DA) as a means of merging observation with model output to improve upon the accuracy of the estimation. This will be explained in detail in section 4.

Some of the most commonly used remote sensing instruments for soil moisture observation are the Multi-Spectral Scanner (MSS), Thematic Mapper (TM), thermal infra-red line scanner, Synthetic Aperture Radar (SAR), and microwave radiometer. Although numerous remote sensing systems are in existence and have been utilized for soil moisture measurement, the most appropriate is microwave remote sensing. Microwave remote sensing provides a means of direct measurement of soil moisture for a range of vegetation cover conditions. Such remote measurement provides the opportunity of observing frequent, global sampling of soil moisture with large spatial resolution. The main advantage of microwave measurements is that they are not affected by cloud cover and variable solar illumination; however, the accuracy in soil moisture estimation is limited to regions with either bare soil or low to moderate amounts of vegetation [46].

The two approaches used in microwave soil moisture measurement are active and passive [31]. In active methods a microwave pulse is transmitted and the backscattering from the object is received and compared with the signal sent to determine the backscattering coefficient. In passive methods, the brightness temperature is measured at microwave length. Different portions of the microwave region of the electromagnetic spectrum known as bands are named by letters. Some of the most commonly used bands in Earth remote sensing are: K (18-27 GHZ), X (8-12 GHZ), C (4-8 GHZ), and L (1-2 GHZ) [31]. The best soil moisture information is provided at very low microwave frequencies (< 6GHZ) owing to the reduced atmospheric attenuation and greater vegetation penetration at lower wavelengths. Most of the studies to date have used the observations within L band at 1.4 GHZ as the signals in this band show the maximum sensitivity to surface soil moisture [46]. Due to the effects of moisture on the dielectric constant and emissivity of soil, microwave measurements are sensitive to soil moisture. In fact, the sharp contrast between the dielectric constants for water (about 80 at frequencies below 5GHZ) and that of dry soil (about 3.5) is what makes measuring soil moisture using low frequency passive microwave radiation possible. This large contrast between the dielectric constants of water and that of dry soil translate into difference of up to 100 K or more in brightness temperature between very dry and wet soils [62, 63].

The Advanced Microwave Scanning Radiometer (AMSR-E) of the Earth Observing System (EOS) is currently used for the global soil moisture mapping [47, 48]. AMSR-E measures radiation at six frequencies in the range 6.9-89 GHZ with dual polarization. At an altitude of 705 km, the antenna scans the upwelling scene brightness temperatures over the globe in two days or less with a swath of 1445 km providing near global coverage. Spatial resolution differs depending on the frequency of radiation; at 6.9 GHZ the spatial resolution provided is 60 km and at 89 GHZ the resolution provided is 5 km. The operational NASA Level-2B AMSR-E “AE_Land” product includes retrievals of surface soil moisture, a vegetation/roughness correction, and quality control variables [47, 49]. Currently the AMSR-E soil moisture algorithm is working based on a change detection approach using the calibrated AMSR-E channel brightness temperatures [49]. AMSR-E soil moisture retrievals are made using the EASE-Grid product. The C-band AMSR-E footprint data (level 2A) used by Jackson et al. [32] with a resolution of approximately 60 km and a sampling resolution on average of 10 km along with the geophysical ancillary data were mapped to an EASE-Grid with 25-km resolution. Within each of these regions ground-based soil moisture sampling was conducted at a minimum of 36 geographically distributed points. Ground sampling included gravimetric soil moisture measurements at a depth of 1 and 6 cm and dielectric probe measurements of the top 6 cm, soil temperatures, surface roughness, and vegetation parameters.

During calibration and validation field campaigns of the Soil Moisture EXperiments in 2002-2004 (SMEX02, SMEX03, and SMEX04) [4, 5, 32] the accuracy of the soil moisture algorithm was investigated on short time scales. Some levels of consistency and calibration stability of the observed brightness temperatures at specific locations were seen in the results. It was concluded, however, that the spatial variability of the retrieved soil moisture over areas with different amounts of vegetation is subject to biases.

## 3. Snow Observations

Fractional snow cover area (SCA) observations can typically be obtained from visible or infrared satellite sensors providing high spatial resolution observations [28]. However, the effectiveness of this type of sensing is limited by cloud conditions. A better alternative are space-borne passive microwave remote sensors which are capable of qualitative observations of snow water equivalent (SWE). Since 1978 several satellites have made passive microwave measurements at snow water equivalent sensitive frequencies [15]:

1) The scanning Multichannel Microwave radiometer (SMMR), a 5 frequency radiometer providing observations from October 1978 to August 1987;
2) The Special Sensor Microwave Imager (SSM/I), providing observations from September 1987 until present; and
3) The Advanced Microwave Scanning Radiometer for the Earth Observing system (AMSR-E), providing observation from May 2002 until present.

The AMSR-E operational snow mapping algorithm employs an empirical relationship to estimate SWE from surface brightness temperature while providing SWE estimate at a spatial resolution of 25 km. The microwave sensors measure snow mass under cloudy and nighttime conditions, however, dense vegetation cover and water bodies cause large retrieval errors [22]. Studies by Dong et al., [15] shows that the SWE retrievals are not sensitive to thin snow packs (SWE <10 mm). Several studies have shown that SCA and SWE observations are good sources of information to improve upon the model snow estimates [1, 8, 55, 57, 58].

Snow Cover Area (SCA) among many other land surface features is available since 1999 as a 500-meter daily gridded product from the Moderate Resolution Imaging Spectroradiometer (MODIS) sensor flown on board the Terra Earth Observing System (EOS) platform [27]. The product provides a binary classification per pixel for snow cover, cloud, or bare ground. Due to improved spectral resolution and higher spatial resolution of the MODIS as compared to GOES and AVHRR, the more accurate SCA can be obtained from MODIS. The study by Maurer et al., [40] demonstrated that MODIS has the ability to significantly better classify the greater amount of snow in topographically complex and forested basins. The MODIS product has been available since February 2000. One of the limitations of MODIS data is cloud cover. Andreadis and Lettenmaier, [1] used a fractional cloud cover threshold of 20% to decide whether to use the observation or not. They assimilated the SCA if less than 20% of the grid cells in their modeling domain were covered by cloud.

## 4. Hydrologic Data Assimilation

An explosion of activities has been witnessed over the past two decades on the development and application of data assimilation systems. Data assimilation is a way to integrate the data from variety of sources with different resolutions and accuracies with model prediction to improve deterministic model accuracy [41]. In other words, data assimilation is used to not only update the hydrological model states that optimally combine model outputs with observation, but also to quantify observational and hydrological model errors. Data assimilation has been used in other disciplines including the ocean and meteorological sciences to improve upon the predictability of short term weather forecasting models. In recent years, development and application of data assimilation in hydrologic modeling has grown with the intention to exploit the increased availability of remotely sensed land surface variables [10, 39, 42, 52, 61]. Numerous studies have also evaluated the assimilation of soil moisture, snow, and surface skin temperature observations [e.g. 1, 6, 10, 16, 39, 51, 52, 55, 57]. These studies demonstrate the potential of data assimilation to improve the land surface model predictions as well as explore the difficulties and complexities in data management associated with data assimilation.

One of the data assimilation techniques that have been used in hydrologic application is the variational method [see 54, 56]. In this method the problem is formulated as a set of model states that minimizes a cost function defining the model residual. The model error in this method is generally assumed to be time-invariant. As noted by Seo et al., [56] the state-space formulation of the system is not needed in this procedure, however, the assumption of time-invariant model covariance is not realistic. The derivation of the adjoint model, which is essentially the linearized hydrologic model, also adds to the complexity of implementation of this procedure.

Among many other data assimilation techniques, the sequential assimilation algorithms using filtering have garnered the attention of hydrologists due to flexibility in handling all sources of uncertainties and as well as the possibility of ingesting the data sequentially as it becomes available. One of the early hydrologic data assimilation methods is the application of linear Kalman filter [e.g., 34, 35]. In the case of nonlinear, hydrological model the data is rendered in state-space form and by assuming that the model state variables are differentiable the Extended Kalman filter (EKF) can be used. By using this method the model error at the time of observation can be estimated by propagating the covariance matrix of model errors. As reported by Evensen [19] and Reichle et al. [53] the EKF can lead to unstable results in the presence of strong nonlinearity in the system. As noted by Reichle et al., [53] EKF cannot be used in large scale environmental assimilation problems such as distributed hydrological models. This problem can be bypassed by ignoring the spatial correlations among variables in the watershed. This assumption, however, highly limits the application of EKF because the knowledge of spatial correlation among the state variables or the model fluxes is of paramount importance for accurately updating the model state variables.

Another approach to data assimilation is Monte Carlo (ensemble) methods. These methods have received considerable attention by hydrologists in recent years as they are easy to implement and the computational burden is less of an issue with the increased computing power nowadays. To cope with the drawbacks of the EKF, a Monte Carlo-based Kalman filter called ensemble Kalman filter (EnKF) was introduced by Evensen [19]. One of the advantages of the EnKF when compared to the standard EKF is that the estimation of priori model covariance is not needed for the updating (analysis) step although its calculation using the model ensemble is straightforward. The primary application of EnKF in hydrology is the soil moisture or soil temperature profile estimation improvement in vertical direction by assimilating in-situ observation or remote sensing data [10, 17, 24, 29, 30, 38, 53]. Moradkhani et al. [43] extended the application of the EnKF to dual state and parameter estimation of conceptual hydrologic models while the time-varying uncertainty of the states and parameters were obtained through this procedure. For some other applications of EnKF in uncertainty assessment of conceptual rainfall-runoff model, please see [63, 64].

The EnKF uses a Monte Carlo approach to approximate the conditional second-order moments of variables of interest using a finite number of randomly generated model replicates. However, the major limitation of all the filtering techniques rooted in Kalman filtering is their closure at the second-order moments implying that the filter evolution is characterized by their model state covariance. Also, the EnKF is limited to the linear updating rule with considerable simplification while using a highly nonlinear hydrologic model. This has encouraged the hydrologists to look into other filtering techniques, such as Particle filter (PF) [44, 64, 65], to avoid the aforementioned limitations. The main difference of particle filter from other data assimilation methods is that the model state variables are not updated but rather their probability distributions are evolved through time. In fact the model ensemble members are characterized by a set of discrete random particles with associated weights (probabilities). The probability distributions of model predictions are then calculated as a weighted combination of the ensemble members [2, 44].

### 4.1. Sequential Bayesian Data Assimilation using Ensemble Filtering

The mathematical framework of estimation theory provides the tools required to approach variety of data assimilation problems. The basic objective of data assimilation is to characterize the state of an environmental system at some future time based on the knowledge of the initial system state. Bayesian inference provides a mechanism to combine the quantitative (hydrologic data) and qualitative data (prior information obtained by the experience of experts in the field) to yield the posteriori as more informative probability distribution of variable of interest. Bayesian formulation allows hydrologists to estimate the uncertainty about model prediction in a systematic way and can be accomplished without resort to calibration which is sometimes problematic in certain applications.

In a Bayesian formulation, the solution to an inverse problem is given by posterior probability distribution *P(M*/*D)* over the model space. *P(M*/*D)* encompasses all the available information of a model which are taken from both data *(D)*, through the likelihood function *P(M*/*D)*, and also data-independent prior information expressed by prior probability *P(M)* density. The mathematical description of Bayes law is given as:

(1) $$P(M\;|\;D)=\frac{P\;(D\;|\;M)\;P\;(M)}{P\;(D)}$$

where the denominator, *p(D)* is the normalization factor. In other words it ensures that the integration of *p(M*/*D)* results to 1. The likelihood function *p(D*/*M)* which measures the likelihood of a given model *M* through its misfit *e(.)*, the residual between observation and model simulation, is given in general form as follows:

(2) p(D|M)∝exp(−e(.))

With the assumption that the model residuals are mutually independent (normally distributed) with constant variance (i.i.d.) the likelihood function can be computed using [7]:

(3) $$p\;(D\;|\;M)\propto \exp\left[-\frac{1}{2}\sum \;{\left(\frac{e\;(.)}{\sigma }\right)}^{2}\right]$$

In the absence of an explicit mathematical expression for *P(D*/*M)* and *P(M)*, which is common in high dimensional problems, Monte Carlo sampling is used to explore posterior *P(M*/*D)*. The importance sampling, Metropolis-Hastings algorithm and Gibbs sampler are the most commonly used sampling techniques in practice. It should be noted that the sampling should not be biased toward any particular region of parameter space and thereby no possibility of entrapment in local minima.

The original Bayes law explained above (eq. 1) is in the batch form where the available historical data is taken for the uncertainty estimation through the conditional probability. However, this form makes no attempt to include information from new observations as they become available. The flexibility required to use the new information is provided by a sequential Bayesian scheme. Moradkhani et al. [43, 44] showed that the methods based on sequential Bayesian estimation are better able to benefit from the temporal organization and structure of information achieving better conformity of the model output with observations.

Let's consider the state variable *x_t_* as the quantity of interest to be estimated within the Bayesian framework. Due to stochastic nature of *x_t_*, the pertinent information about it at any time *t* can be extracted from the observation *Y_t_* = [*y_1_*, *y*_2_, … *y_t_*] through the recursive Bayes law:

(4) $$p\;\left(x_{t}\;|\;Y_{t}\right)=p\;\left(x_{t}\;|\;y_{t},Y_{t-1}\right)=\frac{p\;\left(y_{t}\;|\;x_{t}\right)\;p\;\left(x_{t}\;|\;Y_{t-1}\right)}{p\;\left(y_{t}\;|\;y_{1:t-1}\right)}=\frac{p\;\left(y_{t}\;|\;x_{t}\right)\;p\;\left(x_{t}\;|\;Y_{t-1}\right)}{\int p\;\left(y_{t}\;|\;x_{t}\right)\;p\;\left(x_{t}\;|\;Y_{t-1}\right)dx_{t}}$$

**Figure 1.**
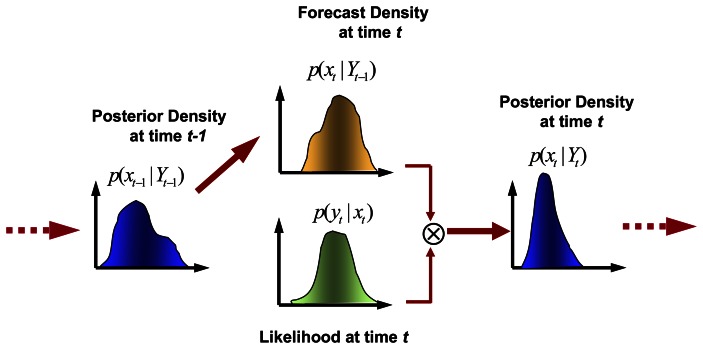
Sequential Bayesian scheme for evolution of the conditional probability density of the state variables by assimilating observations from time *t-1* to time *t*.

As seen in the schematic of recursive Bayes law (see Figure 1 below), the forecast density of *p(x_t_*/*Y_t-1_)* can be estimated via Chapman-Kolmogorov equation [33] assuming that *x_t_* follows the Markov property, therefore:

(5) $$p\;\left(x_{t}\;|\;Y_{t-1}\right)=\int p\;\left(x_{t}\;|\;x_{t-1}\right)p\;\left(x_{t-1}\;|\;Y_{t-1}\right)dx_{t-1}$$

The main complication in using the recursive Bayes law remains in the multidimensional integration of forecast density as shown in eq. (5) which makes the closed form solution of posterior density (eq. 4) practically intractable. This suggests that the ensemble methods through the usage of Monte Carlo sampling provide a practical solution to such problems.

#### 4.1.1. Ensemble Kalman Filter

In sequential filtering, the uncertain state of a hydrological system *x_t,_*, given a set of observation *y_1:t_* is presented by the conditional probability density function *p(x_t_*/*y_1:t_)*. Ensemble methods can be used to calculate the sample approximation to this density function by generating the random replicates of model state variables. Following Jazwinski [33] the generic nonlinear dynamic system in earth sciences are written in discrete-time for both state and measurement equations as follows:

(6) $$x_{t}=f\;(x_{t-1},u_{t},\theta )+w_{t}$$

(7) $$y_{t}=h\;(x_{t})+v_{t}$$

where *x_t,_*is an *n*-dimensional vector of true but uncertain state variables, *u_t_* is a vector of uncertain true of model inputs, *θ* is vector of model parameters and *w_t_* represents the uncertainties due to errors in model formulation, *y_t_* is the measurement vector and *v_t_* is a vector of additive random measurement errors. The model and measurement errors are typically assumed to be Gaussian and independent random vectors with mean zero and covariances *Q_t_* and *R_t_* respectively. Two sequential estimation operations are discerned in filtering applications:

1) the forecasting step which is the transition of state variables from one observation time to the next represented through transition probability *p(x_t_*/*x_t-1_)* in eq. (5),
2) the analysis (updating) step which involves updating of the forecasted (propagated) states with the new observation.

Ensemble procedures present a practical alternative to an exact Bayesian solution by relying on discrete estimation of forecast (priori) and analysis (posteriori) densities through a set of random variables and corresponding weights:

(8) $$p\;\left(x_{t}\;|\;Y_{t-1}\right)\approx \sum_{i=1}^{n}w_{t}^{i-}\delta \;\left(x_{t}-x_{t}^{i-}\right)$$

(9) $$p\;\left(x_{t}\;|\;Y_{t}\right)\approx \sum_{i=1}^{n}w_{t}^{i+}\delta \;(x_{t}-x_{t}^{i+})$$

These are the empirical approximations of forecast and analysis (update) densities by summation of *n* Dirac delta functions where *x^i^* and *w^i^* denote the *i^th^* sample and its weight before and after updating shown by minus and plus signs respectively. The random replicates and associated weights are generated through a variety of methods, one of which is the ensemble Kalman filter (EnKF).

The forecasting step in the EnKF where the evolution of the model for each ensemble member is equally weighted, $w_{t}^{i-}=1/n$, is presented in below:

(10) $$x_{t}^{i-}=f\;\left(x_{t-1}^{i+},\theta ,u_{t}^{i}\right)$$

Thereby, the forecast density in (8) will become:

(11) $$p\;\left(x_{t}\;|\;Y_{t-1}\right)\approx \frac{1}{n}\sum_{i=1}^{n}\delta \;\left(x_{t}-x_{t}^{i-}\right)=\frac{1}{n}\sum_{i=1}^{n}\delta \;\left[x_{t}-f\;\left(x_{t}^{i-},u_{t}^{i},\theta \right)\right]$$

It is noted that in this process, the generation of random input samples of $u_{t}^{i}$ is required to generate the model state replicates in eq. (11). One way to generate the input replicates is to consider the standard error obtained from eq. (7) and generate the random variable using the Gaussian distribution as illustrated in Moradkhani et al. [45]. As seen through eq. (11), the forecasting step is a Monte Carlo approach to derive the *p*(*x_t_* |*Y*_*t*-1_) from the uncertainty in $u_{t}^{i}$ using *p*(*u_t_*) and in some applications by the uncertainty inherent in the parameters of the model through *p*(*θ*). For more details on the inclusion of parameter uncertainty in the filtering, see Moradkhani et al., [43, 44].

If the dynamical system, including states and measurement equations, are linear and all sources of uncertainty are normally distributed the celebrated Kalman filter provides the optimal recursive solution to the state updating problem. If the system is nonlinear, as is the case for most of the hydrologic systems, the linearization of the system might be considered. Developed from the early work using state-space filtering, Georgakakos et al. [25] implemented an automatic procedure into the NWSRFS using the EKF. Certain shortcomings of the procedure have been discovered including reformulation of the original SAC-SMA model to a state-space form, using first order approximation of Taylor series which leads to unstable results when the nonlinearity in the model is strong, and heavy computational demands owing to error covariance propagation. To overcome the limitation of the EKF, the EnKF was introduced Evensen [19] which were used for assimilating data in large nonlinear ocean and atmospheric models. The EnKF is also based upon Monte Carlo or ensemble generations where the approximation of the forecast state error covariance matrix is made by propagating an ensemble of model states using the updated states from the previous time step. The key point in the performance of the EnKF is to generate the ensemble of observations at each update time by introducing noise drawn from a distribution with zero mean and covariance equal to the observational error covariance matrix; otherwise the updated ensemble will possess a very low covariance [43].

Let denote *X*^−^ as the ensemble of forecasted model state (*x_1_*, *x_2_*, …, *x_m_*) at each time *t*, for each of the state variables having *n*-ensemble members, that is

(12) $$X_{t}^{-}=\{x_{t}^{1-},x_{t}^{2-},\ldots ,x_{t}^{n-}\}$$

If the priori error in the forecasted ensemble members is shown by $e_{t}^{-i}=\{x_{t}^{i-}-{\bar{x}}_{t}\}$ with

(13) $$E[x_{t}^{-}]={\bar{x}}_{t}^{-}=\frac{1}{n}\sum_{i=1}^{n}x_{t}^{i-}$$

And, $e_{t}^{-}=\{e_{t}^{1-},\ldots ,e_{t}^{n-}\}$

Then the model error covariance is calculated directly from the ensemble as follows:

(14) $$P_{t}^{-}=E[e_{t}^{-}\;e_{t}^{{-}^{T}}]=\frac{1}{n-1}e_{t}^{-}\;e_{t}^{{-}^{T}}$$

Knowing the priori error covariance of model states from (14), the state updates can be obtained by (15):

(15) $$x_{t}^{i+}=x_{t}^{i-}+K_{t}\;\left(y_{t}^{i}-{\hat{y}}_{t}^{i}\right)=x_{t}^{i-}+K_{t}\;\left(y_{t}^{i}-Hx_{t}^{i-}\right)$$

Where

(16) $${\text{K}}_{\text{t}}={\text{P}}_{\text{t}}^{-}{\text{H}}^{\text{T}}{\left(\text{H}{\text{P}}_{\text{t}}^{-}{\text{H}}^{\text{T}}+\text{R}\right)}^{-1}=C_{t}^{xy}\;{\left[C_{t}^{yy}+R\right]}^{-1}$$

where ${\text{P}}_{\text{t}}^{-}{\text{H}}^{\text{T}}=C_{t}^{xy}$ is the cross covariance of model states and observation prediction, ${\text{HP}}_{\text{t}}^{-}{\text{H}}^{\text{T}}=C_{t}^{yy}$ is the covariance of the observation prediction and H is linearized observation transformation matrix in eq. (7). If it is assumed that the forecast and measurement are jointly normal, their densities are sufficiently characterized by their mean and covariances, meaning that the higher order moments are ignored in the update step.

In the EnKF implementation, the observation *y_t_* at each time should be perturbed, usually using normal distribution with zero mean and variance **R**. This creates an ensemble of perturbed observation which are used in eq. (15) to update the model ensemble members.

#### 4.1.2. Particle Filter

Similar to Ensemble Kalman filter, the sequential Bayesian algorithm can be used to derive the particle filter. Various names are associated with the particle filters such as bootstrap filter, the condensation algorithm, sequential Monte Carlo sampling, interacting particle approximations, and survival of the fittest [2]. Unlike the Kalman filter which simplifies the recursive estimation by assuming Gaussian distribution for state variables, the particle filter relaxes the need for restrictive assumptions regarding the forms of the probability densities; that is, PF can easily manage the propagation of non-Gaussian distribution through nonlinear hydrologic models [44]. To improve the estimation accuracy and stability it is possible to track the time evolution of the model by means of all moment characteristics through a full probability density function [2, 44]. This is facilitated by using particle filters. Particle filters share the same forecasting step with EnKF. However, for the updating step, the updated ensemble members (replicates) are kept the same as the forecast values and only the weights (probabilities) are updated. As mentioned earlier, in PF the state ensemble members are not updated but rather their probability distributions. Therefore,

(17) $$x_{t}^{i+}=x_{t}^{i-}$$

and from eq. (4), the filtering posterior, $w_{t}^{i+}$ is calculated as follows:

(18) $$w_{t}^{i+}=b.p\;\left(y_{t}\;|\;x_{t}^{i+}\right)\;w_{t}^{i-}=b.p\;\left(y_{t}\;|\;x_{t}^{i-}\right)\;w_{t}^{i-}=\frac{b}{n}p\;\left(y_{t}\;|\;x_{t}^{i-}\right)$$

Where $p\;\left(y_{t}\;|\;x_{t}^{i-}\right)$ is the likelihood function for the forecasted replicates, *b* is the normalizing constant in eq. (18), *b* = *1*/*p*(*y_t_* | *y*_1:_*_t_*_−1_) and we defined earlier that $w_{t}^{i-}=1/n$. By substituting eqs. (17) and (18) into eq. (9), we can obtain the update (posterior) probability distribution. In the case of Gaussian likelihood, the problem of degeneracy of particles (ensemble collapse to a single point) may be experienced as those particles that are closer to the measurement get higher weights while others are discarded. One solution is to use many particles which, in the case of a distributed model, may not be a cost effective solution. The second method is to implement the resampling technique to prevent the samples from degeneracy. Some of the sampling techniques used in particle filtering are the Sequential Importance sampling (SIS), Sequential Importance Resampling or Sampling Importance Resampling (SIR) and regularized sampling [2] as the most commonly used sampling procedures. Employing the proper sampling technique keeps the particles from dispersion due to stochastic behavior of the system or degeneracy. For detailed information on SIR-particle filter and sampling see Moradkhani et al. [44]. Through the SIR filter, the resampling is made with replacement n times. In fact, the probability of a selection of any sample of *j* is equal to $w_{t}^{j+}$. When resampling is over, the new ensemble of equally weighted particles of $x_{t}^{j+}$ with weights $w_{t}^{j+}=1/n$ is created. In this process the replicates with higher weights (probabilities) have a higher chance to be selected and the low weight replicates are more likely to be discarded.

After the resampling step, the posterior distribution will be presented as:

(19) $$p\;\left(x_{t}\;|\;Y_{t}\right)\approx \frac{1}{n}\sum_{i=1}^{n}\delta \;\left(x_{t}-x_{t}^{j+}\right)]$$

The new resampled replicates are taken and forwarded in time and procedure through equations (10) and (11) is continued.

#### 4.1.3. DA Experiment Setup through Observing System Simulation Experiment (OSSE)

**Figure 2.**
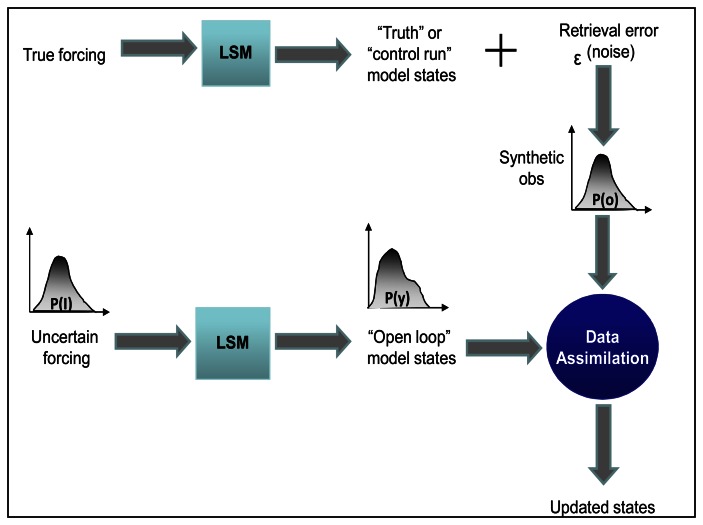
Schematic of Observing System Simulation Experiment (OSSE).

In general, OSSE is designed to enable the modeler to examine the performance of data assimilation procedures and even to obtain the sensitivity of the procedure to different models with different parameterizations and physical representations. OSSE typically consists of at least four components: (1) A simulated data sets of land model states, (2) a forward model to link the states with observations, (3) a model to degrade these observations accuracy and spatial resolution, (4) integrating the degraded observation into a prediction model. A schematic of OSSE is shown in figure 2:

For such synthetic experiments, “truth” is defined when the model is integrated (run) for a set of meteorological, soil, vegetation and initial conditions. Considering that the observation from microwave remotely sensed data for soil moisture are in the form of brightness temperature, the model output (soil moisture) should be converted to brightness temperature to make them ready to be ingested in the updating step of the filtering process. Therefore, the modeled soil moisture is taken to a radiative transfer model (RTM) [47]. By doing so, a synthetic brightness temperature is generated from the model. To account for the measurement noise a zero mean normally distributed random number is added to the brightness temperature which synthetically creates the noisy observation (see figure 2). The open loop simulations are conducted when uncertain inputs are propagated into the model to degrade the model estimate. To ensure that the uncertainty in the model input is realistic a meteorological forcing data set different from that of the truth run is used. For example, as described by Kumar et al. [37], the land surface model is spun using meteorological forcing from the Global Data Assimilation System (GDAS) - the global operational weather forecast model of the National Center for Environmental Prediction (NCEP) [13] creates the “truth” or control run then the open loop simulation is conducted while forcing the land surface model with meteorological forcing from Goddard Earth Observing System (GEOS) [50].

## 5. Summary

This paper provides a review of the most commonly used remotely sensed land surface measurements, mainly microwave remote sensing products for both soil moisture and snow (SWE and SCA) to be used in a data assimilation framework to improve upon the land surface model prediction. Passive microwave remote sensing provides a means of direct measurement of soil moisture for a range of vegetation cover conditions. Such remote measurement provides the opportunity of observing frequent, global sampling of soil moisture with large spatial resolution.

Fractional snow cover area (SCA) observations can typically be obtained from visible or infrared satellite sensors providing high spatial resolution observations [28]. However, cloud conditions are always limiting the effectiveness of these sensors in retrieval. SCA can also be obtained from the Moderate Resolution Imaging Spectroradiometer (MODIS). Studies demonstrate that MODIS has the ability to significantly better classify the greater amount of snow in topographically complex and forested basins. Although MODIS data has shown some limitations to cloud cover, it is suggested as a better product well-suited for data assimilation. Space-borne passive microwave remote sensors can also provide a capability for qualitative observations of snow water equivalent (SWE). Among several satellites that have made passive microwave measurements for snow water equivalent, Advanced Microwave Scanning Radiometer for the Earth Observing system (AMSR-E), was used by [1], however, the magnitude of improvement was found to be minimal as compared to the assimilation of SCA from MODIS.

The concept of data assimilation was discussed and two of the advanced techniques, mainly EnKF and PF were explained in detail. Considering that DA techniques are used mainly for reducing the uncertainty, there is still a lack of consensus in hydrologic community on the selection and implementation of a suitable land DA method to meet this need. It needs to be realized that contemporary DA methods are used for estimating the state variables (here, soil moisture, SWE or SCA), and uncertainties associated with them, however, not all sources of uncertainties are addressed in the assimilation process. These include uncertainty in model parameters and also model structure which are ignored in DA implementations. Although in this paper we did not intend to provide a comprehensive review of all the data assimilation methods, we focused on two of emerging techniques as reported by few studies in section 4. It was mentioned that the ensemble filtering using PF results to full representation of prognostic variable and even parameter probability distributions. The EnKF is limited to the linear updating rule as in the original Kalman filter and also assumption of Gaussian distribution of errors in observation and model. Considering that the soil moisture and snow water equivalent probability distribution significantly change over time and are often non-normal, the existing assumptions in EnKF limit its application in strongly nonlinear hydrologic models. Knowing the potentials of PFs, further implementation of PF as an alternative procedure for operational data assimilation is suggested. The synthetic study through OSSE design as seen in section 4.1.3 is an appropriate procedure to judge about the merits of a certain technique for land data assimilation and the method can be used to adequately quantify and minimize the hydrologic predictive uncertainty while including model parameter uncertainty in the whole scheme.
